# Evaluation of indexing pulse pressure variation during low tidal ventilation: An experimental pilot study

**DOI:** 10.1007/s00101-025-01575-w

**Published:** 2025-09-01

**Authors:** Amelie Zitzmann, Fabian Müller-Graf, Tim Bandorf, Susanne Reuter, Jonas Merz, Paul Frenkel, Brigitte Vollmar, Stephan H. Böhm, Daniel A. Reuter

**Affiliations:** 1 Department of Anaesthesiology, Intensive Care Medicine and Pain Therapy, University Medical Centre of Rostock, Schillingallee 35, 18057 Rostock, Germany; 2 Rudolph-Zenker-Institute for Experimental Surgery, University Medical Centre of Rostock, Rostock, Germany

**Keywords:** Hemodynamic monitoring, Fluid therapy, Preload, Protective ventilation, Mechanical ventilation, Hämodynamisches Monitoring, Flüssigkeitstherapie, Vorlast, Protektive Beatmung, Kontrollierte Beatmung

## Abstract

**Background:**

To reliably assess fluid responsiveness using pulse pressure variation (PPV), tidal volumes (VT) of at least 8 ml/kg of ideal bodyweight are recommended. This contrasts with the current recommendations for lung-protective mechanical ventilation, which advocate VTs between 6 and 8 ml/kg to minimize ventilator-induced lung injury.

**Objective:**

The aim of this study was to analyze whether indexing PPV to certain ventilatory parameters can be a possibility for VT-independent assessment of fluid responsiveness during mechanical ventilation with lower tidal volumes.

**Material and methods:**

Hemodynamic and ventilatory data were collected from eight anesthetized, paralyzed, intubated and mechanically ventilated pigs. Each animal was ventilated with four different VTs (4, 6, 8, and 12 ml/kg) during volume-controlled ventilation, across four intravascular fluid states: normovolemia; hypovolemia induced by bleeding and two stages of fluid resuscitation induced by retransfusion and additional fluid administration. The PPV values were indexed to various ventilatory parameters including VT, plateau pressure (Pplat) and driving pressure (∆P), as well as transpulmonary pressures and composite parameters, such as minute ventilation (MV), mechanical power and mechanical energy.

**Results:**

Indexing PPV to MV (PPV/MV) resulted in values with the smallest variation across different VTs, followed by PPV/VT, PPV/Pplat and PPV/∆P. These indexed parameters exhibited high ratios of explained variance (R^2^) to regression slope (β), indicating reduced VT dependency. In each case, higher values reflected a greater calculated fluid deficit.

**Conclusion:**

Indexing PPV to MV can be a feasible way to use dynamic parameters of fluid responsiveness across a wide spectrum of ventilator settings, such as during lung protective ventilation strategies involving lower tidal volumes. Future studies should evaluate the performance of the indexed parameters in guiding fluid therapy in the clinical setting and define thresholds.

**Graphic abstract:**

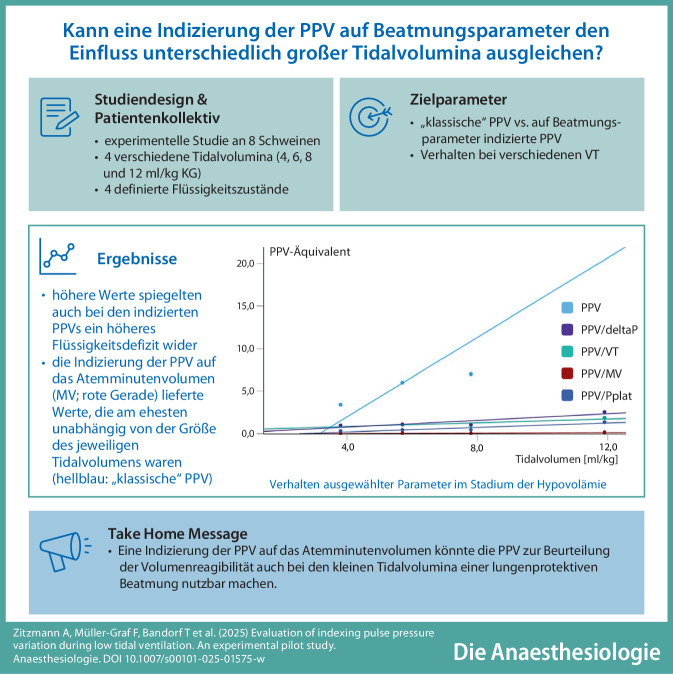

## Abstract (de)

**Hintergrund:**

Zur zuverlässigen Beurteilung der Volumenreagibilität mit Hilfe der sog. Pulsdruckvariation (*pulse pressure variation*, PPV) werden Tidalvolumina (VT) von mindestens 8 ml/kg Idealgewicht empfohlen. Diese Anforderung steht jedoch im Widerspruch zur lungenprotektiven Beatmung, für die VT von 6 bis maximal 8 ml/kg empfohlen werden.

**Ziel:**

Ziel dieser experimentellen Studie war es herauszufinden, ob die Einschätzung der Volumenreagibilität bei künstlicher Beatmung mit niedrigeren Tidalvolumina verbessert werden kann, wenn man die „klassische“ PPV auf verschiedene Beatmungsparameter indiziert.

**Material und Methoden:**

Kreislauf- und Beatmungsparameter von acht narkotisierten, relaxierten, intubierten und kontrolliert beatmeten Schweinen wurden unter volumenkontrollierter Beatmung mit vier verschiedenen VT (4, 6, 8 und 12 ml/kg Idealgewicht) erhoben. Diese Messungen erfolgten bei vier definierten intravasalen Volumenstatus, die durch kontrolliertes Ausbluten, schrittweise Retransfusion und zusätzliche Volumengabe erzeugt wurden. Die jeweiligen PPV-Werte wurden auf verschiedene Beatmungsparameter indiziert: auf das jeweils angewandte Tidalvolumen, auf Plateaudruck (Pplat), *driving Pressure* (∆P) und transpulmonale Drücke sowie auf kombinierte Parameter wie das Minutenvolumen (MV), mechanische Energie (*mechanical energy*) und mechanische Leistung (*mechanical power*).

**Ergebnisse:**

Die Indizierung der PPV auf das Minutenvolumen (PPV/MV) zeigte die geringste Abhängigkeit vom jeweils eingestellten Tidalvolumen. Mehr Abhängigkeit zeigten PPV/VT, PPV/Pplat und PPV/∆P. Höhere Werte der indizierten PPV korrelierten dabei jeweils mit einem größeren kalkulierten Volumendefizit.

**Schlussfolgerung:**

Die Indizierung der PPV auf das Minutenvolumen stellt eine praktisch gut umsetzbare Möglichkeit dar, dynamische Parameter der Volumenreagibilität auch unter lungenprotektiver Beatmung mit niedrigen Tidalvolumina nutzbar zu machen. Diese indizierten Parameter könnten dann ohne Anpassung der Beatmung zur Steuerung der Volumentherapie eingesetzt werden. Ihre klinische Anwendbarkeit und tatsächliche Aussagekraft sollten in weiterführenden Studien überprüft werden.

## Treten Sie in den Austausch

Diese Arbeit wurde für *Die Anaesthesiologie* in Englisch eingereicht und angenommen. Die deutsche Zusammenfassung wurde daher etwas ausführlicher gestaltet. Wenn Sie über diese Zusammenfassung hinaus Fragen haben und mehr wissen wollen, nehmen Sie gern in Deutsch über die Korrespondenzadresse am Ende des Beitrags Kontakt auf. Die Autorinnen und Autoren freuen sich auf den Austausch mit Ihnen.

## Introduction and background

Ventilation-induced changes in the arterial pulse pressure (pulse pressure variation, PPV) are commonly used to predict fluid responsiveness. These parameters are generated by the impact of controlled mechanical ventilation (CMV) on the heart, the pulmonary vasculature and on the major intrathoracic vessels, resulting in changes in cardiac preload and afterload [22]. The magnitude of inspiratory, plateau, driving and alveolar pressures [20, 23, 32] and tidal volume (VT) [1, 7, 26] are known to affect PPV, whereas high respiratory rates [2] and positive end-expiratory pressures (PEEP) [24] can limit their sensitivity. When PPV was introduced into clinical practice more than two decades ago, higher VT were common [25]. In recent years, concern about ventilator-induced lung injury (VILI) [31] has led to a reduction in VT, not only in patients with acute respiratory distress syndrome (ARDS) but also in patients without lung disease [4, 25]. Furthermore, the concept of mechanical power (MP), a composite variable that reflects the energy transferred from the ventilator to the patient over time, was introduced incorporating more potential causes of VILI [13]. The resulting lung protective ventilation strategies, however, reduce the sensitivity and precision of PPV for estimating volume responsiveness with previously accepted cut-off values [22]. Therefore, attempts have been made to change these parameters to different ventilation parameters to maintain their applicability [5, 32, 33].

The aim of this study was to investigate the effects of different tidal volumes on the hemodynamics and on parameters of fluid responsiveness at different fluid states. Furthermore, we indexed PPV to single and composite ventilatory parameters to evaluate its behavior between the different tidal volumes. We hypothesized that indexing PPV would provide parameters independent of the magnitude of VT.

## Study design and investigation methods

The study was approved by the governmental ethics board for animal research (Landesamt für Landwirtschaft, Lebensmittelsicherheit und Fischerei, Rostock, Mecklenburg-Vorpommern, Germany; No. 7221.3-1-059/19; veterinarian in charge: Dr Sylvia Hille) on 29 January 2020 and carried out in accordance with the EU Directive 2010/63/EU and the ARRIVE 2.0 guidelines [10]. Animals were euthanised in compliance with European and federal laws using pentobarbital (45 mg/kg intravenously) while maintaining general anesthesia. Parts of this study have been published before [34].

### Anesthesia and instrumentation

For this study 16 German Landrace pigs (12–16 weeks old) were prepared and anesthetized according to local standards. The animals were brought to the stables of the Institute for Experimental Surgery 4–5 days before the experiment for acclimatization with free access to food and water until the night prior to the study. Each animal’s health was checked by the staff of the Central Laboratory Animal Facility on the morning of the trial and admitted only if it showed no abnormalities as defined by local standards. The pigs were equipped with a 3-lead electrocardiogram (ECG), a pulse oximeter (Nellcor® PM10N, Medtronic, Watford, UK) placed at the tail and two peripheral venous cannulas (20 G) in the earlobes. After induction with 200 µg fentanyl, 100 mg propofol and 4 mg pancuronium, a combination of fentanyl (10 µg/kg h^−1^), propofol (4–8 mg/kg h^−1^), midazolam (0.1 mg/kg h^−1^) and pancuronium (6 mg h^−1^) was used for maintenance of anesthesia to ensure a deep level of sedation and to suppress any spontaneous breathing. Orotracheal intubation was performed with a tube of 7 mm internal diameter and the pigs were mechanically ventilated using the Servo‑u ventilator (Getinge AB, Gothenburg, Sweden) to ensure gas exchange during further instrumentation. For assessment of transpulmonary pressures, a NutriVent® nasogastric tube (SIDAM group, Mirandola, Italy) was placed and connected to the auxiliary pressure port of the ventilator. The position was verified using a positive pressure occlusion test [8]. All pigs received a 4 Charrière (Ch) 16 cm PiCCO® (Getinge AB, Gothenburg, Sweden) catheter via the right femoral artery for transpulmonary thermodilution (TPTD) measurements, a 5 Ch high-fidelity pressure sensor catheter (Mikro-Tip® SPR-350, Millar Instruments Inc., Houston, TX, USA) in the descending aorta via the left femoral artery and a central-venous catheter in the right internal jugular vein for hemodynamic monitoring. For induction of hypovolemia, an 8.5 Ch introducer sheath was placed in the right common carotid artery and another one in the right internal jugular vein for retransfusion. For measurement of pulmonary artery pressures, a 7.5 Ch thermodilution pulmonary artery catheter (Arrow®, Teleflex Inc., Wayne, PA, USA) was placed via the venous introducer sheath. Correct catheter placement was verified by fluoroscopy.

### Fluid status

For comparison of PPV at different hemodynamic conditions, four intravascular fluid states (IVFS) were induced:

- Baseline (BL): after instrumentation, a first set of thermodilutions (TD) was performed to assess the intravascular fluid state (IVFS). If stroke volume variation (SVV) was greater than 10%, repeated boluses of colloids (modified gelatine 4% in Ringer’s acetate solution) of 100 ml each were given until SVV remained < 10%. Another set of TD was performed for confirmation. This fluid state was defined as baseline, intended to represent normovolemia.
- Hypovolemia (Hypo): in this step, 25 ml/kg of blood was withdrawn and collected in transfusion bags with heparin for later retransfusion. In cases of severe hemodynamic instability, blood withdrawal was paused to allow for hemodynamic stabilization, was stopped if considered too dangerous or resumed when deemed appropriate by the principal investigator. Such hemodynamic instability was defined as a decrease in blood pressure or continuous cardiac output measured via the femoral arterial line down to a level at which the PulsioFlex® monitor was no longer able to provide measurements, together with a decrease in end-tidal CO_2_ by more than 50%.
- Resuscitation stage I (Res I): a total of 50% of the previously withdrawn blood was retransfused.
- Resuscitation stage II (Res II): in addition to the remaining half of the withdrawn blood, 20 ml/kg of colloids were infused.

### Ventilation

Aiming at an optimal inflation of the lungs with as few atelectasis as possible to distribute the applied tidal volume evenly within the lungs while achieving the most uniform perfusion without shunt [14, 27, 29], the ventilator’s built-in automatic stepwise recruitment maneuver (Auto SRM) was used increasing PEEP at a constant driving pressure (∆P) of 15 cmH_2_O until an inspiratory pressure of 40 cmH_2_O was reached. This pressure was held over 50 s before decreasing PEEP in steps of 2 cmH_2_O while assessing dynamic respiratory system compliance. Optimal PEEP was defined as the one being 2 cmH_2_O above the closing PEEP. This individualized PEEP was then used for the duration of the protocol. At each IVFS, the same ventilation sequence was performed: after an initial recruitment maneuver, the pigs were ventilated with volume-controlled ventilation (VCV). The VT was first set to 8 ml/kg bodyweight (BW), and respiratory rate (RR) to result in an end-tidal partial pressure of carbon dioxide (etCO_2_) between 4.3 and 5.7 kPa, with an inspiration:expiration ratio (I:E) of 1:2 and an inspiratory pause of 30%. In the next steps, VT was set to 4, 12, and 6 ml/kg BW and the RR was adjusted to maintain etCO_2_ in the desired range and minute elimination of carbon dioxide (VCO_2_) at a constant level, while the I:E ratio and inspiratory pause remained unchanged. At each VT, hemodynamic and ventilatory data were recorded over a 3-min period after a steady state, defined as changes in tidal elimination of carbon dioxide (VtCO_2_) of less than 10% for 1min, was achieved. Finally, inspiratory and expiratory hold maneuvers of 10s each were performed to measure total PEEP, Pplat and to derive ∆P.

### Data recording and processing

Hemodynamic data including ECG were recorded at a sampling rate of 10 kHz using bridge transducer amplifiers in combination with the respective hardware and software PowerLab 16/35 and LabChart 8 (both ADInstruments, Dunedin, New Zealand). Cardiac output was measured via TPTD using the PulsioFlex® system and ventilation data were recorded using the ServoTracker® software (all Getinge AB, Gothenburg, Sweden). In addition, airway pressures (Paw) were measured at the Y‑piece of the breathing circuit with PowerLab 16/35 for synchronization with the hemodynamic data.

At each protocol step recorded data were checked for phases of arrhythmia, which were excluded before representative 1‑min periods of good quality data were chosen for further analysis. Data analysis was performed with LabChart and Matlab (MathWorks®, Natick, MA, USA).

The PPV was calculated as PPV = (max. − min. pulse pressure) / (max. + min. pulse pressure)/2 using a custom built MatLab routine.

For analyzing the energy transferred from the ventilator, mechanical power (MP) was calculated as MP = 0.098 × * RR * VT * (Ppeak − 0.5 * ∆P), as suggested by Chiumello et al. [9]. Mechanical energy per single breath was calculated accordingly as ME = MP / RR.

For indexing PPV for the respective ventilation parameters, the PPVs at the respective fluid state and tidal volume were divided by the corresponding ventilation parameter for each animal. Afterwards, regression analysis was performed.

### Statistics

Statistical analysis was performed using SPSS Statistics version 29 (IBM, Armonk, NY, USA). Hemodynamic and ventilation parameters are displayed as median (interquartile range, IQR). Friedman’s two-way analysis of variance on ranks (ANOVA) was used to test for differences between the fluid states and the tidal volumes, the Tukey test was used for all pairwise multiple comparison procedures and Bonferroni’s correction to compensate for alpha-inflation by multiple testing. *P* ≤ 0.05 was considered statistically significant. For regression analysis, an overall F‑test was performed and parameters with *p*-values > 0.05 were considered as having no explanatory power for the model.

To be considered independent of VT, indexed parameters need to have a low (close to zero) regression factor (β). Moreover, high coefficients of determination (R^2^) were requested for proof of linear regression. To compare the indexed parameters, the ratio of R^2^ to β was calculated for each parameter at each fluid state; higher R^2^/β-ratios indicate higher agreement with these criteria.

## Results

As 8 animals died before completion of the protocol, complete data sets of 8 pigs weighing 40.3 kg (30.8–43.0 kg) were recorded, analyzed and are reported here.

For induction of hypovolemia, 826 ml (425–1069 ml) corresponding to 22.5 ml/kg (14–25 ml/kg) of blood were withdrawn.

### Ventilatory data

Auto SRM determined the optimal PEEP to be 10 cmH_2_O for all but one animal, which had a PEEP of 8 cmH_2_O.

Respiratory rates, airway and transpulmonary pressures at the respective tidal volumes and fluid states are shown in Table 1. Delivered VTs did not differ significantly between the respective fluid states (*p* = 0.757, *p* = 0.259, *p* = 0.516 and *p* = 0.109 for 4, 6, 8, and 12 ml/kg, respectively).

**Table 1:** Ventilation parameters at the respective fluid states

Ventilation parameter	Fluid state	Target tidal volume (ml/kg BW)			
		4	6	8	12
Respiratory rate (1/min)	Baseline	35.0 (32.0–36.8)	30.0 (25.3–30.0)	24.5 (21.0–28.8)	14.0 (10.5–15.8)
	Hypovolemia	35.0 (32.5–35.0)	30.0 (29.3–31.8)	26.5 (20.0–30.0)	14.5 (10.5–15.0)
	Resuscitation I	35.0 (35.0–36.8)	30.0 (27.8–32.0)	26.5 (25.0–30.0)	13.5 (12.0–19.5)
	Resuscitation II	35.0 (35.0–35.0)	30.0 (26.5–31.5)	28.0 (25.0–29.8)	13.0 (12.0–17.3)
	*P value*	*0.519*	*0.887*	*0.202*	*0.643*
Peak inspiratory pressure (cmH_2_O)	Baseline	16.3 (15.6–17.3)	18.8 (18.1–20.2)	21.7 (20.5–24.9)	23.5 (22.8–24.0)
	Hypovolemia	16.0 (15.1–18.4)	20.2 (18.1–22.0)	23.4 (20.7–25.3)	22.9 (21.9–23.7)
	Resuscitation I	16.2 (15.3–18.2)	20.0 (17.6–21.9)	23.1 (20.4–25.0)	23.5 (21.8–25.0)
	Resuscitation II	16.0 (15.2–18.2)	18.8 (17.8–21.7)	23.6 (21.2–25.4)	22.9 (21.8–24.3)
	*P value*	*0.005*	*0.851*	*0.277*	*0.822*
Plateau pressure (cmH_2_O)	Baseline	12.9 (12.0–14.0)	14.5 (13.7–15.2)	15.8 (15.7–17.0)	18.5 (17.5–19.3)
	Hypovolemia	12.8 (11.9–13.5)	14.5 (13.9–15.2)	16.3 (15.4–16.6)	18.4 (17.7–18.7)
	Resuscitation I	12.5 (11.6–13.4)	14.3 (13.4–15.0)	15.6 (14.8–16.4)	17.9 (17.0–18.7)
	Resuscitation II	12.8 (11.9–13.5)	14.3 (13.6–15.0)	16.1 (15.4–16.5)	18.2 (17.4–18.4)
	*P value*	*0.001*	*0.019*	*0.079*	*0.074*
Driving pressure (∆P) (cmH_2_O)	Baseline	4.0 (3.5–4.1)	5.2 (4.7–5.8)	7.0 (5.6–7.5)	9.3 (8.5–9.9)
	Hypovolemia	3.7 (3.4–3.9)	5.3 (4.8–5.8)	6.9 (6.4–7.9)	9.1 (8.4–9.4)
	Resuscitation I	3.6 (3.4–3.9)	5.2 (4.7–5.5)	6.5 (6.1–7.1)	8.8 (8.–9.4)
	Resuscitation II	3.7 (3.3–3.7)	5.0 (4.4–5.4)	7.2 (6.2–7.5)	9.0 (8.–9.2)
	*P value*	0.006	0.058	0.300	0.176
Transpulmonary driving pressure (cmH2O)	Baseline	2.4 (1.7–2.5)	2.8 (2.3–3.6)	3.3 (3.0–4.3)	5.1 (3.7–6.3)
	Hypovolemia	2.5 (1.3–3.0)	3.6 (2.0–4.8)	4.9 (2.9–6.2)	6.4 (3.8–7.8)
	Resuscitation I	2.1 (1.8–2.7)	3.1 (2.2–4.2)	3.8 (2.9–4.8)	5.2 (3.8–6.4)
	Resuscitation II	2.2 (2.0–2.4)	2.9 (2.7–3.1)	4.2 (3.2–5.3)	5.3 (4.5–5.8)
	*P value*	*0.909*	*0.932*	*0.512*	*0.366*
Mechanical power (W)	Baseline	6.8 (5.6–9.6)	8.6 (7.4–14.4)	12.5 (9.4–19.5)	9.5 (9.3–13.5)
	Hypovolemia	6.3 (5.3–8.7)	12.2 (7.2–15.2)	12.7 (9.0–20.1)	9.2 (8.7–10.2)
	Resuscitation I	7.6 (5.3–9.9)	12.9 (6.8–16.6)	14.8 (10.6–19.0)	12.0 (8.9–14.5)
	Resuscitation II	7.6 (5.3–9.7)	11.1 (7.6–16.4)	15.7 (11.7–20.5)	10.0 (8.6–12.3)
	*P value*	*0.252*	*0.736*	*0.392*	*0.392*
Mechanical energy (mechanical power per breath) (J)	Baseline	0.22 (0.16–0.28)	0.35 (0.25–0.48)	0.63 (0.38–0.70)	0.89 (0.60–0.95)
	Hypovolemia	0.20 (0.15–0.27)	0.39 (0.25–0.48)	0.63 (0.37–0.69)	0.85 (0.61–0.94)
	Resuscitation I	0.22 (0.15–0.29)	0.4 (0.24–0.55)	0.63 (0.39–0.72)	0.83 (0.70–0.93)
	Resuscitation II	0.22 (0.15–0.28)	0.38 (0.26–0.55)	0.66 (0.41–0.72)	0.84 (0.64–0.98)
	*P value*	*0.093*	*0.878*	*0.545*	*0.537*
Minute ventilation (ml/kg*min)	Baseline	133 (118–141)	165 (143–178)	182 (164–226)	162 (127–175)
	Hypovolemia	130 (120–135)	173 (161–194)	196 (156–237)	163 (124–171)
	Resuscitation I	133 (130–133)	176 (152–185)	207 (192–235)	156 (142–227)
	Resuscitation II	133 (127–136)	171 (151–183)	222 (186–233)	155 (142–199)
	*P value*	*0.374*	*0.937*	*0.190*	*0.765*

Data are presented as median (IQR). Friedman’s two-way ANOVA by ranks with 3 degrees of freedom; all-pairwise testing with Bonferroni’s correction for multiple tests

### Hemodynamics and transpulmonary thermodilution

Blood withdrawal decreased the global end-diastolic volume index (GEDI) from 462 ml/m^2^ (416–516 ml/m^2^) at baseline to 324 ml/m^2^ (299–343 ml/m^2^) (hypovolaemia) while the first resuscitation step increased it to 366 ml/m^2^ (313–407 ml/m^2^) and the second to 477 ml/m^2^ (437–502 ml/m^2^). The TD-derived baseline cardiac index was 3.32 l/min/m^2^ (2.90–3.74 l/min/m^2^) and decreased to 1.76 l/min/m^2^ (1.39–2.03 l/min/m^2^) in the postexsanguination phase. With the resuscitation maneuvers it increased to 2.40 l/min/m^2^ (1.93–2.80 l/min/m^2^) and 3.84 l/min/m^2^ (3.37–4.08 l/min/m^2^), respectively. The courses of arterial, pulmonary arterial, and central venous pressures during the interventions and at the different tidal volumes together with the respective PPV and SVV values are shown in Table 2.

**Table 2:** Hemodynamic parameters at the respective fluid states

Hemodynamic parameter	Fluid state	Set tidal volume (ml/kg BW)				*p*
		4	6	8	12	
Heart rate (bpm)	Baseline	103 (85–113)	102 (86–113)	109 (89–115)	102 (88–114)	*0.02*
	Hypovolemia	112 (100–150)	116 (102–155)	121 (106–164)	116 (104–151)	*0.03*
	Resuscitation I	97 (90–110)	96 (95–116)	109 (98–120)	101 (94–117)	*0.12*
	Resuscitation II	89 (86–110)	93 (89–111)	93 (88–113)	93 (91–114)	*0.02*
	*P*	*0.03*	0.01	< 0.01	*0.04*	*–*
Systolic pressure (mm Hg)	Baseline	128 (103–138)	121 (101–137)	129 (98–139)	121 (98–136)	*0.10*
	Hypovolemia	70 (64–78)	68 (56–77)	61 (49–64)	61 (57–71)	*<* *0.01*
	Resuscitation I	91 (76–104)	87 (61–97)	89 (66–104)	83 (60–97)	*<* *0.01*
	Resuscitation II	121 (108–125)	114 (103–117)	124 (108–131)	116 (104–118)	*0.08*
	*P*	*<* *0.01*	*<* *0.01*	*<* *0.01*	*<* *0.01*	*–*
Mean arterial pressure (mm Hg)	Baseline	100 (83–111)	94 (80–110)	99 (77–113)	93 (77–103)	*0.03*
	Hypovolemia	55 (51–58)	52 (46–58)	47 (42–50)	48 (45–53)	*<* *0.01*
	Resuscitation I	67 (57–76)	65 (46–71)	58 (42–68)	61 (46–71)	*0.01*
	Resuscitation II	90 (78–94)	84 (75–86)	93 (78–97)	83 (74–86)	*0.07*
	*P*	*<* *0.01*	*<* *0.01*	*<* *0.01*	*<* *0.01*	*–*
Diastolic pressure (mm Hg)	Baseline	86 (73–98)	80 (69–97)	85 (66–100)	78 (66–90)	*0.03*
	Hypovolemia	45 (44–48)	43 (40–49)	39 (35–43)	40 (37–46)	*0.02*
	Resuscitation I	55 (47–62)	53 (39–58)	54 (42–66)	50 (39–59)	*<* *0.01*
	Resuscitation II	71 (62–79)	67 (59–73)	76 (62–80)	67 (59–70)	*0.02*
	*P*	*<* *0.01*	*<* *0.01*	*<* *0.01*	*<* *0.01*	*–*
Central venous pressure (mm Hg)	Baseline	7.0 (4.7–10.0)	6.9 (4.9–10.0)	6.3 (5.3–10.5)	6.9 (5.0–10.1)	*0.39*
	Hypovolemia	6.1 (3.1–8.5)	6.0 (3.2–8.6)	6.2 (3.3–9.0)	5.9 (3.4–8.7)	*0.04*
	Resuscitation I	6.5 (4.0–10.5)	6.5 (3.7–10.2)	6.7 (3.8–11.2)	6.6 (3.8–10.3)	*0.32*
	Resuscitation II	11 (6.6–12.1)	9.6 (6.1–11.6)	10.6 (7.7–13.3)	10.0 (6.3–11.7)	*<* *0.01*
	*P*	*<* *0.01*	*<* *0.01*	*<* *0.01*	*<* *0.01*	*–*
Mean pulm. artery pressure (mm Hg)	Baseline	18.0 (15.7–23.0)	18.5 (15.8–20.7)	18.2 (16.1–21.0)	18.2 (15.7–20.3)	*0.39*
	Hypovolemia	17.1 (14.0–18.3)	17.6 (14.8–20.6)	15.9 (13.1–17.9)	16.9 (14.6–19.6)	*0.08*
	Resuscitation I	19.8 (11.5–21.5)	19.6 (13.8–22.6)	19.1 (11.3–21.7)	19.1 (14.2–20.0)	*0.09*
	Resuscitation II	21.7 (11.8–23.0)	19.5 (12.0–22.2)	22.0 (12.6–24.2)	20.1 (11.4–23.7)	*0.04*
	*P*	*0.26*	*0.79*	*0.08*	*0.29*	*–*
Pulse pressure variation (%)	Baseline	3.0 (2.0–3.3)	5.8 (3.4–6.0)	5.9 (4.2–7.0)	15.8 (13.7–18.2)	*<* *0.01*
	Hypovolemia	5.6 (4.6–7.1)	8.7 (8.4–12.3)	15.1 (11.8–16.4)	37.6 (34.3–44.3)	*<* *0.01*
	Resuscitation I	3.4 (2.7–4.7)	6.0 (5.0–8.1)	7.0 (6.1–10.8)	22.3 (19.2–26.8)	*<* *0.01*
	Resuscitation II	1.8 (1.2–3.5)	2.6 (2.1–4.7)	2.9 (2.2–3.7)	7.6 (3.8–8.1)	*<* *0.01*
	*P*	*<* *0.01*	*<* *0.01*	*<* *0.01*	*<* *0.01*	*–*
Stroke volume variation (%)	Baseline	5.5 (4.0–11.3)	6.0 (4.3–8.0)	6.0 (5.0–14.5)	15.0 (12.0–18.3)	*<* *0.01*
	Hypovolemia	8.5 (5.0–18.0)	10.0 (7.3–15.8)	10.0 (8.0–15.8)	19.0 (10.5–25.0)	*0.12*
	Resuscitation I	4.0 (3.0–6.0)	5.0 (5.0–11.5)	7.0 (4.3–9.0)	16.5 (15.0–18.0)	*<* *0.01*
	Resuscitation II	3.5 (2.0–5.8)	3.5 (3.0–4.8)	2.5 (2.0–4.8)	5.5 (3.3–7.8)	*0.03*
	*P*	*0.08*	*<* *0.01*	*<* *0.01*	*0.01*	*–*

Data are presented as median (IQR). Friedman’s two-way ANOVA by ranks with 3 degrees of freedom; all-pairwise testing with Bonferroni’s correction for multiple tests

### Pulse pressure variation at different tidal volumes and fluid states

**Fig. 1:**
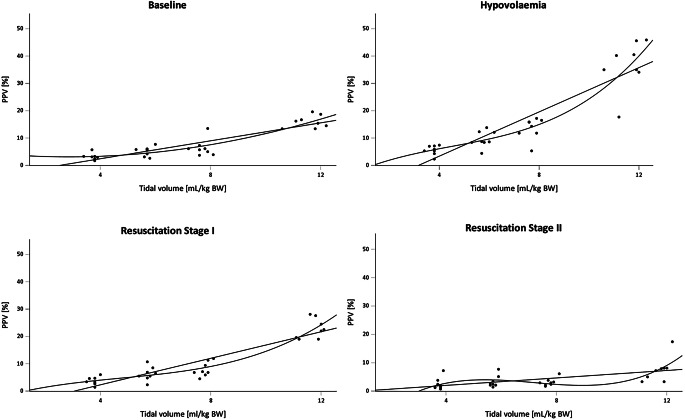
PPV at the respective fluid states and tidal volumes. Regression lines were added with best fit (3rd order) and as linear regression lines with the respective coefficients of determination R^2^ at baseline (BL): 0.842/0.796, R^2^ hypovolemia (Hypo): 0.884/0.819, R^2^ resuscitation stage I (ResI): 0.901/0.833, and R^2^ resuscitation stage II (ResII): 0.516/0.354

The PPV and SVV increased markedly but not significantly after blood removal and dropped again to values below baseline after the second resuscitation step; values at hypovolemia were significantly different from those at resuscitation stage II (*P* *<* *0.01*). Behavior of PPV over the course of the study is shown in Fig. 1 with 3rd order regression curves fitted. Higher tidal volumes resulted in gradually higher values for PPV and SVV at baseline, hypovolemia and resuscitation stage I. The highest values were achieved in hypovolemia, followed by resuscitation stage I and baseline, regardless of tidal volume (Table 3).

**Table 3:** Linear regression analysis of indexed PPV values

Parameter	Fluid state	β	R^2^	R^2^/β ratio	*p (F-test)*
PPV (%)	Baseline	1.644	0.796	0.484	*<0.001*
	Hypovolemia	4.049	0.819	*0.202*	*<0.001*
	Resuscitation I	2.428	0.833	0.343	*<0.001*
	Resuscitation II	0.658	0.354	0.538	*<0.001*
PPV indexed to tidal volume per bodyweight (%/ml/kg BW)	Baseline	0.069	0.289	*4.188*	*0.002*
	Hypovolemia	0.216	0.558	2.583	*<0.001*
	Resuscitation I	0.123	0.481	3.911	*<0.001*
	Resuscitation II	n. a.	n. a.	***–***	*0.983*
PPV indexed to plateau pressure (%/cmH_2_O)	Baseline	0.080	0.801	*10.013*	*<0.001*
	Hypovolemia	0.205	0.775	3.780	*<0.001*
	Resuscitation I	0.129	0.733	5.682	*<0.001*
	Resuscitation II	0.029	0.210	7.241	*0.008*
PPV indexed to driving pressure (%/cmH_2_O)	Baseline	0.123	0.647	5.260	*<0.001*
	Hypovolemia	0.326	0.595	1.825	*<0.001*
	Resuscitation I	0.200	0.579	2.895	*<0.001*
	Resuscitation II	n. a.	n. a.	***–***	*0.814*
PPV indexed to transpulmonary driving pressure (%/cmH_2_O)	Baseline	n. a.	n. a.	***–***	*0.642*
	Hypovolemia	0.571	0.342	0.599	*<0.001*
	Resuscitation I	0.342	0.474	1.386	*<0.001*
	Resuscitation II	n. a.	n. a.	***–***	*0.583*
PPV indexed to mechanical power (%/Watt)	Baseline	0.142	0.591	4.162	*<0.001*
	Hypovolemia	0.409	0.701	1.714	*<0.001*
	Resuscitation I	0.208	0.418	2.010	*<0.001*
	Resuscitation II	0.050	0.129	2.580	*0.044*
PPV indexed to mechanical energy (%/Joule)	Baseline	n. a.	n. a.	***–***	*0.077*
	Hypovolemia	2.548	0.234	0.092	*0.009*
	Resuscitation I	n. a.	n. a.	***–***	*0.170*
	Resuscitation II	n. a.	n. a.	***–***	*0.580*
PPV indexed to minute ventilation (%/ml/kg*min)	Baseline	0.011	0.641	**58.273**	*<0.001*
	Hypovolemia	0.027	0.723	**26.778**	*<0.001*
	Resuscitation I	0.015	0.664	**44.267**	*<0.001*
	Resuscitation II	0.004	0.286	**71.500**	*0.002*

Data from linear regression analysis of PPV and the respective indexed PPV values against tidal volume. β: regression factor; R^2^: coefficient of determination; R^2^/β ratio: higher values reflect a better agreement with the specifications set for the (indexed) parameter (high coefficient of determination, low regression factor); n. a.*: *not applicable for parameters that failed the F‑test (*p* > 0.05)

Please note the low R^2^ values for resuscitation stage II for the applicable parameters

**Fig. 2:**
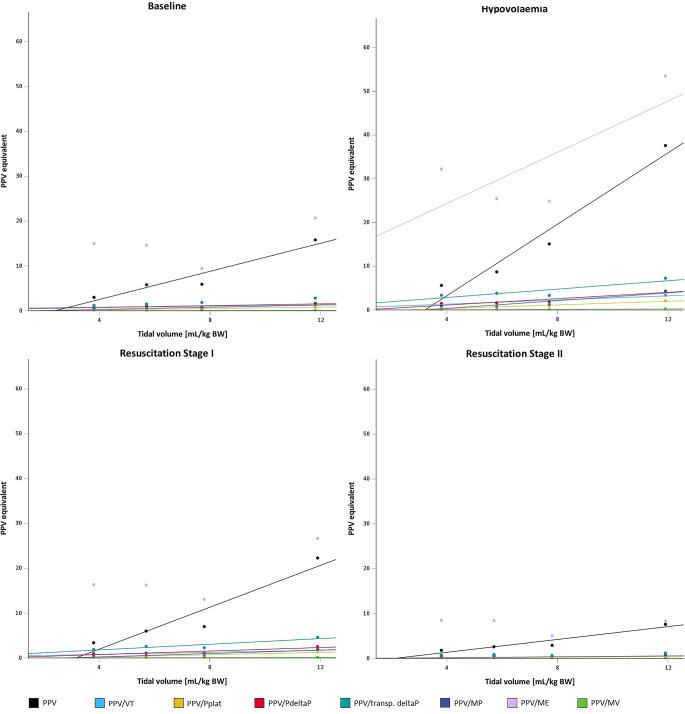
Linear regression lines of the indexed parameters compared to PPV. Filled circles represent the median of PPV (*black*) and the indexed PPV values at the investigated tidal volumes (4, 6, 8, and 12 mL/kg) at the respective fluid states. The gradients of the color-coded lines show the ability to correct for the respective parameter: a flatter curve means a better compensation for the different tidal volumes applied. For parameters that failed the F‑test, no regression lines have been plotted. The Y‑axis has absolute values, but no units, as these are not uniform due to the indexing

Results of linear (1st order) regression analysis (regression factor β, coefficient of determination R^2^, and results of F‑test) of PPV and the corresponding indexed values (PPV/VT, PPV/Pplat, PPV/deltaP, PPV/transpulmonary deltaP, PPV/MV, PPV/MP, and PPV/ME) are listed in Table 3 together with the R^2^/β ratios. PPV indexed to minute ventilation (PPV/MV) provided the highest R^2^/β ratio at each fluid state. Median values of PPV and the corresponding indexed values are presented in Fig. 2 together with linear regression curves.

## Discussion

Blood withdrawal led to physiologically explainable changes in heart rate, cardiac output, arterial pressures and volumetric (i.e. GEDI) as well as in the dynamic preload parameters (i.e. SVV and PPV) [6, 15, 16, 26, 28, 30]: heart rate and SVV and PPV rose, while cardiac output, arterial pressures and GEDI dropped during hypovolemia. Subsequent retransfusion increased arterial pressures but even at ResII, a condition with a positive net fluid balance, they did not reach baseline values, possibly due to some degree of inflammation, nitric oxide production, and/or altered vascular response to endogenic vasoconstrictors [19]. Cardiac index (CI) increased after each fluid bolus until ResII, which contradicts myocardial depression due to hemorrhagic shock as described by Evans and Boyes [12]. Tidal volumes had significant influence on these parameters: heart rate tended to be lower while arterial pressures tended to be higher at lower tidal volumes, especially at the hypovolemic state. From the data presented, ventilation with lower tidal volumes in hypovolemic states could be beneficial for hemodynamics. In our case, higher respiratory rates did not seem to negatively affect cardiac output, as postulated in the work by Szold et al. [30].

The PPV values gradually increased with increasing tidal volumes and driving pressures as described before [1, 7, 20, 23, 26, 32]; however, at the lower VTs (4 and 6 ml/kg BW), values were markedly lower, even when commonly used thresholds for indicating fluid responsiveness were reached at the higher VTs. Indexing PPV to certain ventilatory parameters has been proposed to improve the predictive power of PPV for fluid responsiveness, particularly under low tidal volume ventilation [11]. A linear relationship between PPV and tidal volumes has been described [21] and is the key to the concept of improving the predictive power of PPV under low tidal ventilation by indexing to ventilation parameters. This linearity was demonstrated not only for VT but also for other parameters like PPV/Pplat, PPV/deltaP, PPV/MP and PPV/MV, by high coefficients of determination (R^2^) in our study. Therefore, we reasoned that indexing PPV to ventilatory pressures might also help to interpret the dynamic parameters of fluid responsiveness independently of the magnitude of tidal volume and the controlled or resulting airway pressures. Furthermore, as respiratory rates do not only alter intrathoracic pressure fluctuations but also the times for venous return, we also included composite parameters that include respiratory rate, such as minute ventilation (MV) or mechanical power (MP) to compensate for these factors on the magnitude of PPV, which might not be as direct as VT or driving pressures [2, 11]. A second condition for VT-independent dynamic parameters of fluid responsiveness would be regression coefficients close to zero in the linear regression analysis, indicating that the differences in the parameters between the respective tidal volumes are small and that these are almost cancelled out by the indexing. Again PPV/Pplat, PPV/MP and PPV/MV showed good results. For overall comparison of the indexed parameters with each other and with the genuine PPV, the R_2_/β ratio was used. Considering the single ventilation parameters, indexing PPV to plateau pressure (PPV/plat) had the most favorable combination of regression coefficients close to zero with high coefficients of determination (R^2^; representing high quality of the statistical model), followed by VT (PPV/VT) and airway driving pressure (PPV/∆P). As previously described, PPV values are somewhat proportional to tidal volume [33] and airway driving pressures [23]; however, ventilatory pressures depend on lung und thorax compliance which also affect PPV [17]. This might explain why the impacts of the absolute and relative pressures and the VT on PPV were different. Referring to the study by Liu et al. [20], indexing PPV to transpulmonary driving pressures was not successful in compensating for different tidal volumes in our setting. This may be due to the fact that only a very small proportion of the airway pressures is transmitted to the pleura and therefore differences in the transpulmonary driving pressures were very small between the respective tidal volumes rendering them susceptible to errors due to the blunted transmission [17, 18]. In contrast, the differences in PPV at the respective tidal volumes, especially at lower fluid states, were large; indexing them to small and similar pressures (almost irrespective of the tidal volume) aggravates the differences between the VTs.

Indexing PPV to both tidal volume and respiratory rate, thus to minute ventilation (PPV/MV), resulted in the highest R_2_/β ratio. Thus, this indexed parameter would compensate best for the impact of different tidal volumes. Albeit PPV being calculated over one respiratory cycle, respiratory rate is known to influence the ability of PPV to predict fluid responsiveness, especially if the heart rate-to-respiratory rate-ratio is low [2]. Heart rates tended to be lower at lower tidal volumes but the respiratory rate was increased to compensate for ventilatory minute volume. Therefore, the quotient was even lower, thus reducing the sensitivity for correct assessment of fluid responsiveness even more. Assuming that PPV is caused by the energy transferred from the ventilator, we also considered the combination of tidal volume, pressures, and respiratory rate and thus mechanical power as a potential indexing parameter; however, this resulted in poor correlations, both for PPV/MP and PPV/ME, which statistically failed to show an explanatory contribution.

Indexing PPV to ventilatory parameters generally resulted in parameters with less VT-related fluctuations at the higher fluid states, i.e. resuscitation stage II > baseline > resuscitation stage I > hypovolemia. This can be explained by the overall higher values of PPV at the hypovolemic state with greater differences between the tidal volumes compared to the higher fluid states, where values were lower and differences between the tidal volumes less marked.

### Limitations

We investigated adolescent animals without any known cardiovascular or pulmonary pathology. In our study, the healthy lungs showed a near linear relationship between tidal volume and the respective driving pressures. Therefore, the results we obtained may not be comparable to those studies investigating PPV at lower tidal volumes in ARDS where pulmonary compliance is reduced [1, 32]. Furthermore, this protocol did not use vasopressors to compensate for hypovolemic hypotension as clinicians would normally do to bridge the time until enough fluids have been administered. As we wanted to investigate physiological responses of PPV to different ventilation regimens and fluid states without exogenously induced changes in arterial compliance, vasopressors were omitted.

The question of fluid responsiveness could not be explicitly addressed in this setting as the number of surviving pigs was too small to perform a reasonable ROC analysis. The question whether these indexed parameters can predict an increase in cardiac index after volume administration independently of tidal volume should be investigated in further studies.

As the PiCCO™ technology is not validated for pigs, only parameters directly derived from the thermodilution curves are applicable. Parameters indexed to body surface area (such as CI or GEDI) or parameters calculated with human-related constants can only be used as trends.

## Conclusion

- Indexing PPV to ventilatory parameters can be a way to apply these parameters to assess fluid responsiveness during mechanical ventilation with lower tidal volumes as these indexed parameters better reflect low volume status in these conditions, compared to PPV alone.
- In our experimental setting, PPV indexed to minute ventilation (PPV/MV) resulted in values that could be considered VT-independent and at the same time showed good correlations at all fluid states and tidal volumes.
- As minute ventilation can easily be obtained from the ventilator, this facilitates utilization in clinical practice.
- No specific changes to the ventilation regimen need to be made for hemodynamic assessment.

## Data Availability

Raw data are available on request from the research team.

## Declarations

The study was approved by the governmental ethics board for animal research (*Landesamt für Landwirtschaft, Lebensmittelsicherheit und Fischerei*, Rostock, Mecklenburg-Vorpommern, Germany; No. 7221.3-1-059/19; veterinarian in charge: Dr Sylvia Hille) on 29 January 2020.
